# Amygdala response to emotional faces following acute administration of psilocybin in healthy individuals

**DOI:** 10.1016/j.nsa.2023.103934

**Published:** 2023-12-30

**Authors:** Sophia Armand, Kristian Larsen, Martin K. Madsen, Brice Ozenne, Katrin H. Preller, Gitte M. Knudsen, Dea S. Stenbæk, Patrick M. Fisher

**Affiliations:** a Neurobiology Research Unit and NeuroPharm, Copenhagen University Hospital Rigshospitalet, Denmark; b Department of Psychology, University of Copenhagen, Denmark; c Department of Public Health, Section of Biostatistics, University of Copenhagen, Denmark; d Department of Psychiatry, Psychotherapy and Psychosomatics, University of Zurich, Switzerland; e Department of Clinical Medicine, University of Copenhagen, Denmark; f Department of Drug Design and Pharmacology, University of Copenhagen, Denmark

## Abstract

The serotonergic psychedelic psilocybin acutely induces changes in emotional states. However, it remains unresolved whether psilocybin acutely modulates amygdala reactivity to emotions, a brain region critically involved in emotion processing. Using functional magnetic resonance imaging (fMRI), we examined in 26 healthy individuals whether amygdala responses to angry, fearful and neutral faces differ between acute exposure to psilocybin and at baseline. We also evaluated whether plasma psilocin levels (PPL) and subjective drug intensity (SDI) during psilocybin are related to amygdala responses to the emotional faces. We found that amygdala response to angry faces was significantly reduced during exposure to psilocybin as compared to baseline (mean difference = −0.54, P_FWER_ = 0.03), whereas no significant changes in amygdala responses to fearful or neutral faces were observed. We further found that the amygdala response to fearful faces was significantly negatively associated with SDI (slope = −0.13, P_FWER_ = 0.04), whereas no significant association with PPL was observed. Our findings indicate that psilocybin attenuates amygdala reactivity to angry faces and that a more intense subjective psilocybin response (SDI) is associated with attenuated amygdala reactivity to fearful faces, in accordance with previously reported results. Future studies should investigate whether exposure to psilocybin acutely changes emotion processing in individuals with depression and whether such changes are related to therapeutic outcomes.

## 1 Introduction

Psilocybin is a psychedelic compound gaining renewed attention as a therapeutic to treat various psychiatric conditions when combined with psychological support (Moreno et al., 2006; Anderson et al., 2020; Ross et al., 2016; Bogenschutz et al., 2015, 2022; Garcia-Romeu et al., 2019; Grob et al., 2011; Vollenweider and Preller, 2020; Studerus et al., 2011; Johnson et al., 2019; Carhart-Harris et al., 2018, 2021; Davis et al., 2021; Griffiths et al., 2016). Once ingested, psilocybin is metabolised into psilocin, which dose-dependently induces psychoactive effects by stimulating brain serotonin (5-HT) 2A receptors (5-HT_2A_R) (Vollenweider et al., 1998; Passie et al., 2002; Madsen et al., 2019) over the course of 4-8 h (Stenbæk et al., 2021). The unfolding of the psychoactive effects can be described in three successive phases: ascent, peak and descent (Stenbæk et al., 2021), as measured with subjective drug intensity (SDI). SDI is highly correlated with plasma psilocin levels (PPL) sampled along the psychedelic experience (Madsen et al., 2019). Notably, both SDI and PPL map on to 5-HT_2A_R occupancy (Madsen et al., 2019). Together, SDI and PPL represent two feasible metrics of acute behavioural and neurobiological effects of psilocybin (Madsen et al., 2019; Stenbæk et al., 2021; Madsen and Knudsen, 2021).

A medium to high dose (i.e., >0.2 mg/kg) of psilocybin can dose-dependently induce profound changes in consciousness including changes in emotional state (Vollenweider and Preller, 2020; Studerus et al., 2011; Kometer et al., 2012). According to retrospective self-reports from patients and healthy individuals, psilocybin can occasion emotions such as deep-felt love and peacefulness (Griffiths et al., 2006), emotional breakthroughs (Griffiths et al., 2006; Roseman et al.; Roseman et al., 2019), emotional acceptance rather than avoidance (Watts et al., 2017), increased emotional empathy (Pokorny et al., 2017), as well as re-experiencing of autobiographical emotional memories (Carhart-Harris et al., 2012; Healy, 2021). Thus, from self-reports it seems clear that psilocybin has the capacity to acutely change one’s emotional state, but it remains unclear in what way psilocybin may modulate processing of emotions in the brain. Investigating the neural correlates of emotional processing under the influence of psilocybin using functional Magnetic Resonance Imaging (fMRI) may contribute to our understanding of how the brain processes emotional stimuli during psilocybin intervention.

The amygdala is a brain region critically involved in processing emotions (LeDoux, 2000; Davis and Whalen, 2001; Rhodes et al., 2007; Janak and Tye, 2015), particularly important for detecting the salience and social relevance of threat-related information in the environment (Davis and Whalen, 2001; Haxby et al., 2002; Fusar-Poli et al., 2009). The amygdala response to emotional information is shaped by brain 5-HT signalling (Robinson et al., 2013; Bocchio et al., 2016; Fisher et al., 2015), including 5-HT_2A_R (Fisher et al., 2009, 2011; Hornboll et al., 2013). Only one fMRI study has investigated emotional processing in the amygdala during acute 5-HT_2A_R stimulation with psilocybin compared to placebo, reporting that the amygdala response was lower when viewing negative and neutral scenes (Kraehenmann et al., 2015). A previous study using lysergic acid diethylamide (LSD), another potent psychedelic with a high affinity for the 5-HT_2A_R (Nichols, 2016), found reduced processing of negative faces relative to neutral faces in the amygdala following acute administration of LSD (Mueller et al., 2017). Facial expressions are argued to be clear and pronounced sources of emotional valence (Carrera-Levillain and Fernandez-Dols, 1994), representing prototypic stimuli to investigate emotional processing (Cowen et al., 2019; Said et al., 2011). Yet, it remains to be investigated how psilocybin modulates amygdala reactivity to facial expressions.

Here, we evaluate the acute effects of a medium-high dose of psilocybin on the amygdala response to angry, fearful, and neutral faces. We also examine whether the amygdala response to angry, fearful, and neutral faces is related to PPL and SDI. We hypothesised that 1) amygdala response to angry and fearful faces but not to neutral faces is reduced during acute psilocybin intervention compared to without the drug, 2) PPL and SDI are negatively associated with the amygdala response to angry and fearful faces, but not to neutral faces.

## 2 Methods and materials

### 2.1 Participants

Data were collected from 28 healthy individuals in the study. Data presented here was part of a single-blind cross-over study design wherein participants received either a single 0.2-0.3 mg/kg dose of psilocybin (mean ± SD dose: 19.7 ± 3.6 mg, administered in units of 3 mg capsules) or 20 mg of ketanserin (5-HT_2A_R antagonist) on two separate intervention days separated by at least 21 days. Psilocybin API was synthesized by BAFA UCT Prague (https://bafa.vscht.cz/) and encapsulated by Gentofte Apotek (GMP final medicinal product). As we are primarily interested in psilocybin effects herein, data from the baseline and psilocybin intervention scans are presented here. Participants were recruited from a list of volunteers interested in participating in neuroscientific psychedelic research. Before obtaining their written informed consent, the participants were informed about the study, including side effects and risks. All participants underwent a screening procedure comprising a screening for neurological or significant somatic illness and a screening interview for present or previous psychiatric conditions using a Danish translation of the Mini-International Neuropsychiatric Interview, version 6.0.0 (Sheehan et al., 1998). See the Supplementary text exclusion criteria.

The ethics committee approved this study for the Capital Region of Copenhagen (journal identifier: H-16028698, amendments: 56023, 56967, 57974, 59673, 60437, 62255) and the Danish Medicines Agency (EudraCT identifier: 2016-004000-61, amendments: 2017014166, 2017082837, 2018023295). The study was conducted according to the World Medical Association Declaration of Helsinki and to the Committee on Publication Ethics’ International Standards for Authors.

### 2.2 Experimental design

At baseline, all participants completed the emotional faces paradigm during blood oxygen level dependent (BOLD) fMRI (see detailed description of study outcomes below). We also assessed the participants' baseline intelligence quotient (IQ) using the Reynolds Intellectual Screening Test (Raines et al., 2018), body mass index (BMI), mood using the Major Depression Inventory (MDI) (range from 0 to 50, where >21 indicates a depressed mood) (Bech et al., 2015), sleep quality using the Pittsburgh Sleep Quality Index (PSQI) (range: 0–21, where 45 indicates sleep disturbances) (Buysse et al., 1989) and stress level using the Cohen's Perceived Stress Scale (PSS) (range: 0–40, no cut-off adapted to indicate stress).

Before the day of the psilocybin intervention, participants met with two assisting psychological staff members to prepare for the psilocybin experience. The same staff members facilitated the participant's psilocybin experience with psychological support during the intervention. On the psilocybin intervention day, before dosing, we obtained urine samples from the participants testing for common drugs of abuse (Rapid Response, BTNX Inc., Markham, Canada) and the PSQI, MDI and PSS were completed. In a private room next to the scanner, the participants were given oral psilocybin in gelatine capsules, with a glass of water. We acquired multiple resting-state fMRI and cerebral blood flow (arterial spin labelling) scans before and after the emotional faces paradigm (see (Madsen et al., 2021; McCulloch et al., 2023) for a description of resting-state acquisitions). We aimed to acquire the emotional faces paradigm approximately 150 min after psilocybin administration, however, the acquisition time varied due to timing of the preceding scan sessions and due to participant readiness (Table 1). Prior to and between MRI scans, participants were situated in an adjacent and private room. PPL and SDI assessments took place within the scanner environment. When the psychoactive effects had decreased (i.e., at the end of the day), participants completed the 11-dimension Altered States of Consciousness questionnaire (11D-ASC) (Studerus et al., 2010). The day after the intervention, participants met with the psychological staff members to facilitate integration of the experience. The median interval between baseline MRI scans and the psilocybin intervention was 33 days (range = [5152], IQR = [19, 76]). The participant with a 152-day gap between sessions was due to COVID-19 lockdown. Notably, participants receiving psilocybin as the second intervention necessarily had more than 21 days between baseline and psilocybin intervention scans due to the time between interventions.

**Table 1:** Descriptive information of study sample.

	**Baseline**		**Psilocybin**	
**Categorical variables**	**Frequency**	**Percent**	**Frequency**	**Percent**
N	26		20	
Female/male	9/17	35%/65%	9/11	45%/55%
MRI1/MRI2	12/14	46%/54%	8/12	40%/60%
**Continuous variables**	**Mean ± SD**	**Median [Min; Max]**	**Mean ± SD**	**Median [Min; Max]**
Age (years)	33 ± 8	31 [23; 58]	32 ± 8	29 [23; 58]
Body mass index (kg/m2)	23 ± 3	23 [19; 29]	23 ± 3	23 [19; 29]
**Psychometrics**	**Mean ± SD**	**Median [Min; Max]**	**Mean ± SD**	**Median [Min; Max]**
IQ	113 ± 5	112 [107; 125]	–	–
Percieved stress scale	9 ± 4	9 [1; 16 ]	8 ± 3	8 [1; 13]
Depressive symptoms	5 ± 3	4 [2; 12]	5 ± 3	5 [0; 11]
Sleep quality	4 ± 2	4 [2; 8]	4 ± 2	3 [2; 6]
Altered state of consciousness	–	–	38 ± 15	41 [9; 59]
**Emotional face paradigm**	**Mean ± SD**	**Median [Min; Max]**	**Mean ± SD**	**Median [Min; Max]**
Amygdala response to angry faces	0.55 ± 0.55	0.42 [-0.14; 1.87]	−0.003 ± 0.59	0.02 [-1.30; 0.93]
Amygdala response to fearful faces	0.33 ± 0.39	0.32 [-0.31; 1.15]	0.13 ± 0.58	0.09 [-0.90; 1.34]
Amygdala response to neutral faces	0.28 ± 0.45	0.19 [-0.46; 1.51]	0.30 ± 0.54	0.24 [-0.70; 1.53]
Accuracy for angry faces	0.99 ± 0.03	1 [0.83; 1]	0.96 ± 0.12	1 [0.5; 1]
Accuracy for fearful faces	1.00 ± 0.00	1 [1; 1]	0.97 ± 0.12	1 [0.5; 1]
Accuracy for neutral faces	0.98 ± 0.05	1 [0.83; 1]	0.94 ± 0.15	1 [0.5; 1]
Accuracy for shapes	0.97 ± 0.03	0.97 [0.9; 1]	0.96 ± 0.11	1 [0.5; 1]
Reaction time for angry faces (ms)	1053 ± 190	999 [780; 1483]	1339 ± 322	1319 [899; 1824]
Reaction time for fearful faces (ms)	970 ± 192	936 [611; 1365]	1213 ± 321	1181 [831; 1909]
Reaction time for neutral faces (ms)	1000 ± 163	993 [678; 1379]	1399 ± 437	1312 [697; 2293]
Reaction time for shapes (ms)	936 ± 113	942 [708; 1258]	1175 ± 313	1106 [606; 2101]
Timing of paradigm (min. since drug administration)	–	–	169 ± 24	182 [131; 207]
Plasma psilocin level in μg/L	–	–	12.0 ± 4.2	11.4 [4.9; 20.9]
Subjective drug intensity	–	–	7.15 ± 2.5	8 [0; 10]

Outcomes: IQ and body mass index were measured upon inclusion. On both MRI scan days before initiating scanning, we measured the participants' perceived stress scale with Cohen’s Perceived Stress Scale, major depressive disorder with Major Depression Inventory and sleep quality with Pittsburgh Sleep Quality Index. Amygdala responses to faces is constrasted to geometric shapes. At the end of the psilocbyin intervnetion day, we measuerd the participants' altered state of consciousness with the 11-dimension Altered States of Consciousness questionnaire. Abbreviations: ms = milliseconds, SD = standard deviation, IQ = intelligence quotient, mcg/L = microgram per liter.

### 2.3 Study outcomes

#### 2.3.1 Emotional faces paradigm during BOLD fMRI

In the emotional faces paradigm, which has been used previously (Hariri et al., 2000; Fisher et al., 2022), participants were presented with a trio of faces expressing the same emotions on a screen: one target face in the top centre and two potential matching targets on the bottom left and right (see Supplementary Fig. 1). Participants were instructed to match the target faces as fast and accurately as possible using a glove box (Psychological Software Tools, Pittsburgh, USA). The paradigm consisted of four blocks of emotional faces (i.e., fearful faces, angry faces, surprised faces and neutral faces presented in random order across four versions) interleaved by five control blocks consisting of geometric shapes (i.e., blocks of circles and vertical and horizontal ellipses). Within each block, six face trios were displayed for 4 s, interleaved by a fixation cross (“+”) displayed with variable time intervals (i.e., 2, 4, or 6 s). The geometric shape block followed the same procedure, except that the fixation cross was displayed with a fixed time interval of 2 s. Faces and shapes blocks were preceded by the text “Match Faces” or “Match Shapes,” respectively, presented for 3 s. We included 3 females and 3 males from each emotion category. The paradigm and behavioural responses were recorded using E-Prime (Psychological Software Tools, Pittsburgh, USA). The paradigm was viewed on a mirror that showed the stimuli projected onto a mesh screen mounted at the back of the MRI bore. The paradigm took a total of 6.5 min to complete. To align with previous acute psychedelic studies, which focused on negative and neutral emotional stimuli, we analysed only data from the angry, fearful, and neutral faces as well as shapes conditions (Kraehenmann et al., 2015; Mueller et al., 2017).

#### 2.3.2 Magnetic resonance imaging acquisition parameters

Participants were scanned on one of two 3T Siemens Magnetom Prisma scanners (Erlangen, Germany) using either a 64-channel head/neck coil (MRI_1_) or a 32-channel head coil (MRI_2_) (see participant distribution across scanners in Table 1). All participants completed their two MRI scans (i.e., during baseline and psilocybin) on the same scanner. High-resolution, whole-brain, T1-weighted MPRAGE structural scans were acquired in all participants (MRI_1_: inversion time = 900 ms, repetition time = 1900 ms, echo time = 2.58 ms, flip angle = 9°, in-plane matrix = 256x256 mm, in-plane resolution = 0.9x0.9 mm, 224 slices, slice thickness = 0.9 mm; MRI_2_: inversion time = 920 ms, repetition time = 1810 ms, echo time = 2.41 ms, flip angle = 9°, in-plane matrix = 288x288 mm, in-plane resolution = 0.8x0.8 mm, 224 slices, slice thickness = 0.8 mm). BOLD fMRI scans were acquired during the emotional faces paradigm using a T2*-weighted gradient echo-planar imaging (EPI) sequence (MRI_1_: TR = 2000 ms, TE = 30 ms, flip angle = 90°, in-plane matrix = 64x64 mm, in-plane resolution = 3.6x3.6 mm, 32 slices (interleaved, bottom-up) slice thickness = 3.0 mm, gap between slices = 0.75 mm, in-plane acceleration factor GRAPPA = 2, number of volumes acquired = 195; MRI_2_: TR = 800 ms, TE = 37 ms, flip angle = 52°, in-plane matrix = 104x104 mm, in-plane resolution = 2x2 mm, 72 slices (interleaved, bottom-up) slice thickness = 2 mm, no gap, number of volumes acquired = 488, multi-band acceleration factor = 8). All scans were visually inspected to identify spatial artefacts.

#### 2.3.3 Plasma psilocin levels and subjective drug effects during psilocybin intervention

Immediately before completing the emotional faces paradigm, we 1) asked participants to verbally rate their SDI (“How intense is your experience right now?”) on a Likert scale from 0 to 10 (i.e., from 0 = “not at all intense” to 10 = “very intense”), and 2) acquired a blood sample from an intravenous catheter to measure free unconjugated psilocin (μg/L) plasma level.

### 2.4 Missing data

Some individuals had missing or invalid data for the emotional faces paradigm. This included two participants’ data at baseline (n = 1 due to poor vision; n = 1 due to claustrophobia at baseline only) and eight participants’ data at the psilocybin intervention (n = 1 due to poor vision; n = 2 due to technical errors; n = 4 due to nausea and/or challenges regarding the psychological state; n = 1 participant reported closed eyes during the paradigm). Therefore, data from 26 participants were included in the final analyses. One participant had a 50% accuracy (chance) on the emotional faces paradigm during psilocybin intervention. We included data from this participant as it appeared this person had processed the emotional faces, despite not performing the task as instructed. For PPL, two participants had missing data just before the emotional faces paradigm; in those cases, we used the PPL measure acquired immediately after the paradigm.

### 2.5 Data analysis

#### 2.5.1 Pre-processing and data analyses of fMRI data

See Supplementary Text for additional details on pre-processing of fMRI data. Framewise displacement based on estimated motion parameters and temporal signal to noise ratio (tSNR) was computed to probe sequence and intervention related differences in signal quality. In single-subject general linear models (GLMs), we employed a canonical hemodynamic response function to the smoothed functional images to estimate task-specific BOLD activity (i.e., beta images), including motion parameters and censored volumes. A high-pass filter (128 s) was applied to control for slow frequency fluctuations. The AR(1) and FAST auto-regressive functions were used for MRI_1_ (TR = 2000 ms) and MRI_2_ (TR = 800 ms), respectively (Corbin et al., 2018). The GLMs were used to generate contrast images for our effects of interest (i.e., angry, fearful and neutral faces versus geometric shapes). For each participant, mean BOLD fMRI response to each face condition was extracted from a bilateral amygdala region of interest (ROI). We focused on the regional response of the amygdala to limit the penalty for multiple comparisons.

#### 2.5.2 Quantification of plasma psilocin level

Plasma psilocin level (PPL) was quantified using ultra-performance liquid chromatography and tandem mass spectrometry (measuring free and unconjugated psilocin), as previously described (Madsen et al., 2019). PPL was reported in μg/L.

#### 2.5.3 Statistical analysis

To align with previously reported acute effects of psychedelics, we evaluated the amygdala responses to fear, angry, and neutral faces. To evaluate the amygdala response to angry, fearful, and neutral faces during psilocybin intervention compared to baseline (i.e., scan type: baseline vs. psilocybin intervention), we fitted a linear mixed model (LMM) for each emotion type with an unstructured residual covariance pattern using the packages LMMstar in R (v. 4.0.3). The LMM was used as it accounts for a) repeated measurements within participants, b) different residual variances at the two time points, and c) incorporates all data, even if an individual scan session for a participant is missing. MRI scanner was included as a covariate due to differences in acquisition parameters between MRI_1_ and MRI_2_, as described in section 2.3.2. Age, sex and censored volumes (accounting for motion) were considered covariates. However, due to our small sample size and lack of evidence for associations between amygdala response and age, sex, and censored volumes, these covariates were excluded from final models. In post-hoc analyses using the same statistical model, we assessed effects of the emotional faces paradigm on whole-brain regions during psilocybin compared to baseline.

To evaluate the associations between amygdala response to angry, fearful, and neutral faces and PPL and SDI, respectively, we fitted LMMs for each emotion, again with an unstructured residual covariance pattern and MRI scanner as a covariate. Two additional mean parameters were introduced in the LMM encoding the association between (i) scan time, i.e., baseline vs. psilocybin, and (ii) mean-centred PPL/SDI with amygdala response. Mean-centred values were computed by setting baseline PPL/SDI values at 0, while the mean PPL/SDI was subtracted from individual PPL/SDI values from the psilocybin MRI. These parameters can be interpreted as (i) the change in amygdala response for a person with mean PPL/SDI and (ii) how the amygdala response changes along with PPL/SDI.

We used LMMs (with an unstructured residual covariance pattern) to examine whether there were differences in accuracy and reaction time for the emotional faces paradigm as well as in censored volumes for scans acquired during baseline compared to during the psilocybin intervention.

P-values are reported both uncorrected (punc) and corrected for family-wise error rate (P_FWER_) using the Bonferroni correction method for a family of three tests (i.e., angry, fear and neutral) (Dunn, 1961), as indicated. P-values were considered statistically significant at P_FWER_ < 0.05. P-values for tests of differences in accuracy and reaction time across scan sessions were corrected for a family of four tests (i.e., angry, fear, neutral and shapes). For post-hoc whole brain analyses, p-values were corrected for 88 tests, reflecting the number of brain regions examined.

## 3 Results

### 3.1 Baseline characteristics

Demographics, psychometrics, PPL and SDI at the time of the emotional faces paradigm and behavioural outcomes are summarised in Table 1. MDI scores showed low levels of "depressive symptoms" at baseline (score range = 2–12) and psilocybin intervention (score range = 0–11), the PSQI showed an absence of sleep disturbances by cut-off >45 at baseline (score range = 2–8) and psilocybin intervention (score range = 2–6). The PSS showed low or average stress levels at baseline (score range = 1–16) and psilocybin intervention (score range = 1–13) (Cohen and Janicki-Deverts, 2012). The participants' mean IQ was slightly higher than the average of 100 in the general population (mean ± SD = 113 ± 5). At baseline, we observed a pronounced response of the amygdala to each of the emotional faces contrasted with geometric shapes (see Table 1).

### 3.2 Change in amygdala and behavioural response to the emotional faces paradigm

Fig. 1 displays images of the average amygdala response to each emotion and Fig. 2 displays individual and average and individual amygdala responses at baseline and during psilocybin intervention adjusting for the effect of scanner. Estimated mean parameters are reported in Table 2. The amygdala response to angry faces was significantly decreased during psilocybin intervention compared to baseline (mean difference [95% CI]: 0.54 [−0.94; −0.14], P_FWER_ = 0.03). The amygdala response to fearful faces was numerically decreased during psilocybin intervention, but this effect was not statistically significant (mean difference [95% CI]: 0.21 [−0.52; 0.10], P_FWER_ = 0.50). Amygdala response to neutral faces was only slightly numerically increased during psilocybin intervention and was not statistically significant different (mean difference [95% CI]: 0.02 [−0.22; 0.42], P_FWER_ = 1.00).

**Fig. 1:**
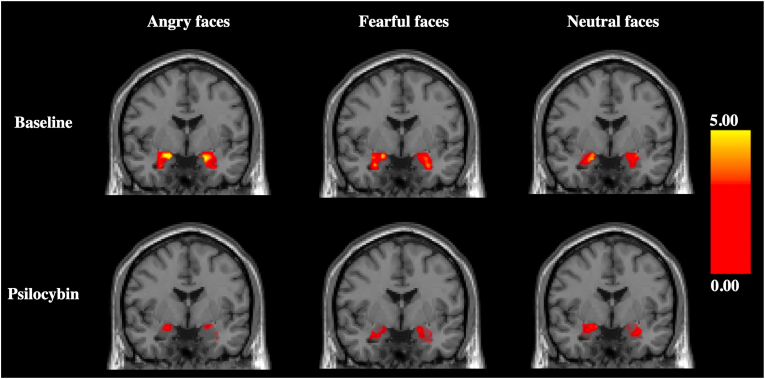
Illustration of amygdala response to angry, fearful, and neutral faces (contrasted with geometric shapes) at baseline (N = 26) and psilocybin (N = 20) sessions (Punc <0.05). Coronal brain slices represented in standard space at Y = −2. Colour bar indicates t-scores.

**Fig. 2:**
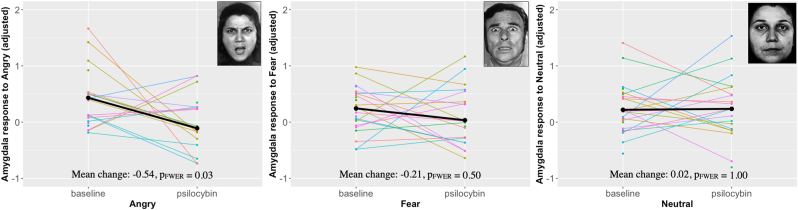
Plots of the partial residuals for the amygdala response (corrected for scanner) to angry faces (left), fearful faces (middle) and neutral faces (right), contrasted to geometric shapes at baseline (n = 26) and during psilocybin intervention (n = 20). Examples of face stimuli are portrayed in each corresponding plot. The black dots represent the means, and the coloured lines represent individual values. To evaluate amygdala response to faces during psilocybin compared to baseline, we used a linear mixed model for each emotion, which showed (mean difference [CI], P_FWER_): Angry: 0.54 [-0.94; −0.14], P_FWER_ = 0.03; fear: 0.21 [-0.52; 0.10], P_FWER_ = 0.50; neutral: 0.02 [-0.22; 0.42], P_FWER_ = 1.00.

**Table 2:** Psilocybin effects on amygdala responses.

	**Estimate**	**SE**	**95% CI**		**punc**	**pFWER**
**Angry Faces**						
Intercept	0.43	0.12	0.18	0.68	0.001	–
**Psilocybin**	**−0.54**	**0.20**	**−0.94**	**−0.14**	**0.010**	**0.031**
MRI	0.21	0.13	−0.07	0.49	0.138	0.414
**Fearful Faces**						
Intercept	0.24	0.11	0.28	0.46	0.028	–
Psilocybin	−0.21	0.15	−0.52	0.10	0.168	0.503
MRI	0.17	0.13	−0.11	0.45	0.221	0.663
**Neutral Faces**						
Intercept	0.22	0.12	−0.03	0.47	0.079	–
Psilocybin	0.02	0.14	−0.28	0.31	0.908	1.000
MRI	0.10	0.15	−0.22	0.42	0.516	1.000

Psilocybin effects expressed with baseline session as reference, i.e., a negative "Psilocybin" effect reflects lower amygdala response during the psilocybin session. MRI scanner effects expressed with MRI2 as reference. "Estimate" is the unstandardised regression coefficient. Abbreviations: SE = standard error of regression coefficient, 95% CI = 95% confidence interval, punc = unadjusted significance level, pFWER = p-values corrected for family-wise error rate using Bonferroni.

The accuracy for the emotional faces paradigm did not differ between exposure to psilocybin and baseline (angry: P_FWER_ = 0.34; fearful: P_FWER_ = 0.98; neutral: P_FWER_ = 0.39; shapes: P_FWER_ = 0.96). Reaction time was numerically slower across all stimuli types during exposure to psilocybin, statistically different for angry and neutral faces (mean difference [95% CI], P_FWER_: angry: 273 ms [149; 398], P_FWER_ < 0.001; neutral: 370 ms [138; 602], P_FWER_ = 0.01), but not for fearful faces and shapes (mean difference [95% CI]: fearful: 182 ms [−11; 376], P_FWER_ = 0.25; shapes: 192 ms [13; 372, P_FWER_ = 0.15).

### 3.3 Associations between amygdala response, plasma psilocin level and subjective drug intensity

**Fig. 3:**
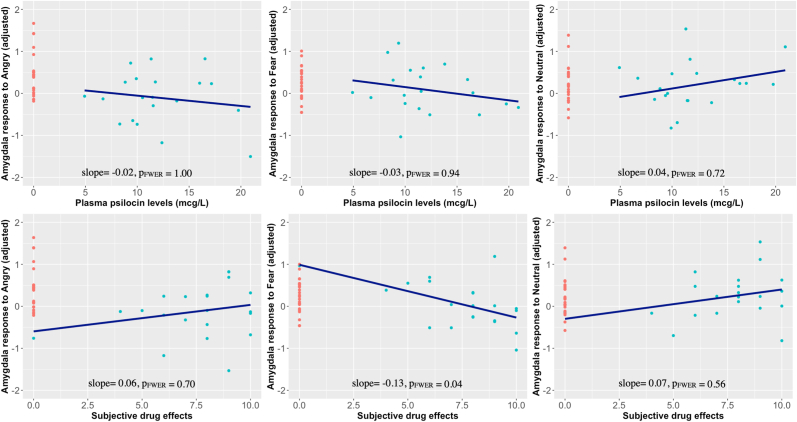
Plots of the amygdala response to angry, fearful and neutral faces (contrasted to geometric shapes) by plasma psilocin levels (PPL)/subjective drug intensity (SDI). Associations were estimated using linear mixed effects models (LMM) with MRI scanner as a covariate. In the LMMs, two additional mean parameters were introduced, encoding the association between (i) scan time, i.e., baseline vs psilocybin, and (ii) mean centred PPL/SDI with amygdala response (i.e., baseline PPL/SDI values were set to 0 and, while the mean PPL/SDI was subtracted from individual PPL/SDI values from the psilocybin MRI). These parameters can be interpreted as (i) the change in amygdala response for a person with mean PPL/SDI and (ii) how the amygdala response changes along with PPL/SDI. Red dots correspond to PPL/SDI at baseline, and blue dots correspond to PPL/SDI at psilocybin. The blues lines correspond to the estimated association between amygdala responses and PPL/SDI. P_FWER_ = corrected for family-wise error rate using the Bonferroni method with three tests.

We found no evidence of a dose-response effect of PPL on amygdala response to angry faces (P_FWER_ = 1.00), fearful faces (P_FWER_ = 0.94) or neutral faces (P_FWER_ = 0.72) (see Supplementary Table 1 for model parameter estimates). We did see a significant negative association between SDI and amygdala response to fearful faces (slope [95% CI]: 0.13 [−0.22; −0.03], P_FWER_ = 0.04) (see Supplementary Table 2 for model parameter estimates). We did not find evidence for an intensity-response effect for angry (P_FWER_ = 0.70) or neutral faces (P_FWER_ = 0.56). Fig. 3 shows associations between amygdala responses and PPL and SDI.

### 3.4 Change in whole-brain response to the emotional faces paradigm

Post-hoc analyses of whole-brain responses to angry and fearful faces, showed that almost all 88 regional brain responses were numerically reduced during acute effects of psilocybin compared to baseline, but no effects were statistically significant after correction for multiple testing (Supplementary Tables 3 and 4). For neutral faces, most brain regions were unchanged or numerically increased from baseline to psilocybin, but no effects of increase were statistically significant after controlling for multiple testing (Supplementary Table 5).

### 3.5 Difference in censored volumes, framewise displacement and tSNR

As observed elsewhere, participants moved more during psilocybin sessions compared to baseline. The framewise displacement was significantly higher in psilocybin scans compared to baseline scans (mean difference [95% CI]: 0.18, [0.035, 0.318], p = 0.017; units: framewise displacement). We observed a very small and not statistically significant difference in framewise displacement between scanners (mean difference [95% CI]: 0.001, [−0.034, 0.037], p = 0.94). We found that there were significantly more censored volumes for scans acquired during psilocybin compared to baseline (mean difference in % of volumes censored [95% CI]: 7.4 [3.7; 11.1], p < 0.001), and a small but significant difference between scanners, such that slightly more volumes were censored on MRI_1_ (mean difference in % of volumes censored [95% CI]: 1.1 [0.1; 2.1], p = 0.04). We observed significantly lower temporal signal-to-noise ratio (tSNR) during the psilocybin sessions (mean difference [95% CI]: 51.6, [−75.7, −27.4], p < 0.001). We did not observe a significant effect of scanner on tSNR (mean difference [95% CI]: 8.7 [−32.5, 15.5], p = 0.47).

## 4 Discussion

We find that amygdala response to angry faces was significantly reduced during acute effects of psilocybin compared to baseline. There were no significant changes in the amygdala responses to fearful or neutral faces, although amygdala response to fearful faces was numerically reduced compared to baseline. We observed a significant negative association between SDI and amygdala response to fearful faces, meaning that a more intense psychedelic experience is associated with greater reduction in fear processing. Our findings show that amygdala response to emotional faces changes during acute effects of psilocybin, which is consistent with previous self-reports describing substantial changes in emotional orientation during a psilocybin experience.

As hypothesised, we find that the amygdala response to angry faces was significantly reduced during psilocybin intervention, but, inconsistent with our hypothesis, we find that the amygdala response to fear was not statistically significantly affected. However, our results showed that the mean amygdala response to fearful faces was numerically reduced, which was not the case for the response to neutral faces, which remained more or less unchanged from baseline to psilocybin. Our result partially aligns with a previous fMRI study investigating amygdala responses to negative and neutral scenes during psilocybin intervention (Kraehenmann et al., 2015). This study found that amygdala responses to both negative and neutral scenes (e.g., neutral pictures of humans, animals or daily activities contrasted to shapes) during psilocybin intervention were significantly lower compared to placebo (Kraehenmann et al., 2015). Neutral stimuli can be emotionally ambiguous and hence the difference between Kraehenmann et al. and our results may reflect divergence in the type of neutral stimuli used in the studies. Another fMRI study investigated amygdala processing of emotional faces following acute LSD administration (Mueller et al., 2017), reporting significantly reduced amygdala responses to fearful faces relative to neutral faces, as compared to placebo. In sum, current evidence shows that acutely following psychedelic administration with psychological support, the amygdala is less responsive to negative faces and negative scenes, which together suggests a reduced sensitivity or attribution of salience to negative stimuli. This reduced sensitivity to negative stimuli has also been demonstrated in studies using cognitive testing (Kometer et al., 2012), EEG (Schmidt et al., 2013) and self-report questionnaires (Studerus et al., 2011). One study in patients with treatment-resistant depression observed increased right amygdala reactivity to fearful and happy faces and no change in left amygdala reactivity one-day following psilocybin administration (Roseman et al., 2018). Future studies scanning a single cohort both during the acute psychedelic experience and afterwards would be informative in resolving these discrepant findings.

Our post-hoc analyses revealed that there was a general numeric reduction of response to angry and fearful faces in almost all brain regions during psilocybin, while responses to neutral faces were generally similar or numerically increased during psilocybin compared to baseline. Although none of these effects were statistically significant after controlling for multiple testing, these results point to an overall reduced brain response to negative emotions during exposure to psilocybin. We speculate that the lack of statistical significance might be due to properties of the emotional faces paradigm as a measurement tool, including unsatisfactory signal-to-noise ratio resulting in noisy estimates of neural activity during emotional processing, in line with previous reports of poor test-retest reliability of the emotional faces paradigm (Plichta et al., 2012). Future studies evaluating effects of psychedelics on emotional processing should consider applying or developing paradigms with a better signal-to-noise ratio to more accurately estimate neural activity during emotional processing.

As hypothesised, we found a negative association between SDI and the amygdala response to fearful faces. In line with our results, a previous study of emotional processing during exposure to LSD reported that higher subjective drug effects were associated with reduced amygdala response to fearful faces (Mueller et al., 2017). Previous studies have found that SDI during acute effects of psilocybin is significantly associated with resting-state brain network integrity and segregation (Madsen et al., 2021), baseline 5-HT_2A_R (Stenbæk et al., 2021) and 5-HT_2A_R psilocin occupancy (Madsen et al., 2019). Our current observations reinforce that SDI is an informative, yet simple and feasible tool with minimal disruption to the participants' psychedelic experience, which can be used to obtain a measure of subjective effects in real time during psychedelic exposure.

### 4.1 Methodological considerations and limitations

For the emotional faces paradigm, we observed a pronounced amygdala response to the task (i.e., to each emotion of interest) at baseline, confirming that the task induced the anticipated neural response. The amygdala responses were comparable to other healthy individuals in the same environment (Fisher et al., 2022). We do not suspect that the observed change in amygdala response to angry faces stems from habituation from baseline to psilocybin because a previous test-retest study at the time scale of weeks did not report such effects (Plichta et al., 2012). Although participants were blind to the receipt of psilocybin in the current study, we suggest that future studies include a placebo or low-dose group to better control effects related to potentially confounding factors, e.g., unblinding or expectancy (Muthukumaraswamy et al., 2021). Participants generally completed the task with a high degree of accuracy across all task conditions and scan times, suggesting that the reduced amygdala response is not a result of less engagement in the task during psilocybin intervention. We found higher reaction times across emotions during the psilocybin intervention, in line with previous findings (Kraehenmann et al., 2015; Mueller et al., 2017; Carter et al., 2005). Higher reaction times are perhaps due to greater cognitive demands related to the completion of the task during the psychedelic experience. The time of scanning (∼3 h after drug administration), represents the turn of peak to descent phase for most of the participants, which is also reflected in the relatively lower PPL and SDI levels in the current study compared to previously reported levels during peak (Madsen et al., 2019; Stenbæk et al., 2021). Consequently, we might have underestimated the effects of psilocybin on emotional face processing, which may be of greater magnitude during peak as seen with other psilocybin-induced neural changes (Madsen et al., 2021).

Due to practical constraints beyond our control, participants were subjected to scanning using one of two 3 T Siemens Prisma MRI scanners, with each individual's scans being completed on a single scanner. While this approach ensured consistency, it also introduced potential heterogeneity in our data stemming from differences between scanners. To mitigate this, we incorporated the MRI scanner type as a covariate in all our analyses. Furthermore, we assessed the tSNR in the amygdala and evaluated the differences between scanner environments, which showed comparable tSNR values. Additionally, considering the susceptibility of multiband EPI sequences to head movement, we evaluated the effects of scanner on framewise displacement, which also showed a non-significant difference between scanner environments.

Although enabling, e.g., higher spatial resolution, multiband sequences are susceptible to distinct types of artefacts vis-à-vis single-band sequences, e.g., slice-leakage and intra-slice aliasing (Todd et al., 2016; McNabb et al., 2020). We cannot rule out that using two different sequences (e.g., multiband vs. single-band; TR = 800 ms vs. 2000 ms, native voxel resolution = 2 mm isotropic with no gap vs. 3.6x3.6x3 mm with gap) increased signal variance and reduced statistical power to detect session effects and related associations. Previous studies have reported reduced tSNR in deep brain structures in multiband acquisitions (Demetriou et al., 2018). We did not observe significant differences in tSNR nor censored volumes between scanners. However, decreased tSNR and increased motion and number of censored volumes during psilocybin sessions suggests a potential negative bias in signal quality, a general challenge in scanning participants during the acute psychedelic experience. Effects of motion on amygdala responses were adjusted for in single-subject GLMs to limit the effects of movement. Further, across all our fitted statistical models, we found that number of censored volumes was not significantly associated with amygdala responses.

In our analyses of association between PPL and amygdala responses to emotional faces, we did not observe significant effects for any emotion. However, we used a two-parameter statistical model, which we speculate might have been too conservative considering our smaller sample size. Future studies should examine dose-response effects in a placebo-controlled study design to establish that PPL does not modulate amygdala responses to emotion.

### 4.2 Conclusion

As hypothesised, we found that the amygdala response to angry faces was significantly reduced but remained unchanged to neutral faces using BOLD fMRI following acute administration of psilocybin in healthy individuals. Consistent with our hypothesis, we also find that amygdala response to fearful faces is negatively associated with SDI. This is the first study to establish that psilocybin acutely modulates amygdala response to angry faces and that the amygdala response to fearful faces is associated with subjective drug intensity. Future studies should investigate whether emotion processing is altered in individuals with depression following acute administration of psilocybin, and whether such alterations are related to long-term therapeutic and clinical outcomes.

## Source of funding and conflicts of interest

The work was supported by The Innovation Fund Denmark (grant ID 4108-00004B), Danish Council for Independent Research - Medical Sciences (grant ID 6110-00518B), Ester M. og Konrad Kristian Sigurdssons Dyreværnsfond (grant number 850–22–55,166–17-LNG) and Rigshospitalet’s Research Council (grant number R130- A5324). SA was supported by 10.13039/501100003554Lundbeck Foundation (grantID R281-2017-4366 and R281-2018-131). DSS was supported by the Danish Council for Independent Research (grant ID 9058-00017B).

MKM has received an honorarium as a speaker for Lundbeck Pharma and the Lundbeck Foundation. KHP is currently an employee of Boehringer-Ingelheim GmbH & Co KG. GMK has received honoraria as a consultant for Sanos and as a speaker for Sage-Biogen. DSS has received an honorarium as a speaker for the Lundbeck Foundation. The remaining authors have disclosed that they have no potential conflicts, including financial, consultant, institutional or other relationships, which could lead to bias or conflict of interest.

## Figures and Tables

**figs1:**
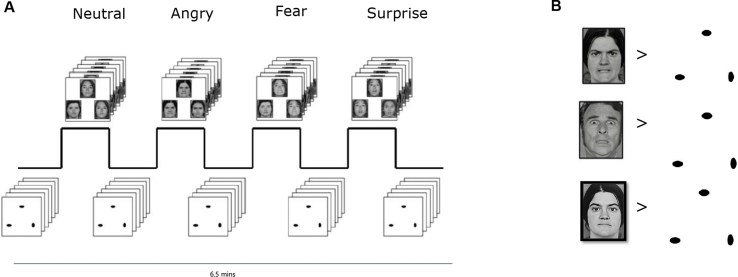
A. Illustration of the emotional faces paradigm during fMRI. Participants are instructed to match the target stimuli (in the top) to one of the two potential matching stimuli on the bottom left and right sides as fast and accurately as possible using a hand-controller pressing the left or right key. The entire paradigm consists of four blocks of emotional faces (i.e., fearful faces, angry faces, surprised faces and neutral faces presented in random order across four versions) interleaved by five control blocks consisting of geometric shapes. Within each block, six face trios were displayed for four seconds, each interleaved by a fixation cross displayed with variable time intervals (i.e., two, four or six seconds) to minimise expectancy effects and habituation while maximising amygdala response throughout the paradigm. For shapes, fixation cross was displayed with a fixed time interval of two seconds, interleaving each of the six shape trios. The paradigm takes a total of 6.5 minutes to complete. B. In this study, we only attended to negative facial expressions (i.e. anger and fear) and neutral faces, as well as geometric shapes, which were used to contrast each facial expression.

## References

[bib1] Anderson B.T. (2020). Psilocybin-assisted group therapy for demoralized older long-term AIDS survivor men: an open-label safety and feasibility pilot study. E Clin. Med..

[bib2] Bech P., Timmerby N., Martiny K., Lunde M., Soendergaard S. (2015). Psychometric evaluation of the major depression inventory (MDI) as depression severity scale using the LEAD (longitudinal expert assessment of all data) as index of validity. BMC Psychiatr..

[bib3] Bocchio M., McHugh S.B., Bannerman D.M., Sharp T., Capogna M. (2016). Serotonin, amygdala and fear: assembling the puzzle. Front. Neural Circ..

[bib4] Bogenschutz M.P., Forcehimes A.A., Pommy J.A., Wilcox C.E., Barbosa P.C.R., Strassman R.J. (2015). Psilocybin-assisted treatment for alcohol dependence: a proof-of-concept study. J. Psychopharmacol. Oxf. Engl..

[bib5] Bogenschutz M.P. (2022). Percentage of heavy drinking days following psilocybin-assisted psychotherapy vs placebo in the treatment of adult patients with alcohol use disorder: a randomized clinical trial. JAMA Psychiatr..

[bib6] Buysse D.J., Reynolds C.F., Monk T.H., Berman S.R., Kupfer D.J. (1989). The Pittsburgh Sleep Quality Index: a new instrument for psychiatric practice and research. Psychiatr. Res..

[bib7] Carhart-Harris R.L. (2012). Implications for psychedelic-assisted psychotherapy: functional magnetic resonance imaging study with psilocybin. Br. J. Psychiatry J. Ment. Sci..

[bib8] Carhart-Harris R.L. (2018). Psychedelics and the essential importance of context. J. Psychopharmacol. Oxf. Engl..

[bib9] Carhart-Harris R. (2021). Trial of psilocybin versus escitalopram for depression. N. Engl. J. Med..

[bib10] Carrera-Levillain P., Fernandez-Dols J.-M. (1994). Neutral faces in context: their emotional meaning and their function. J. Nonverbal Behav..

[bib11] Carter O.L., Burr D.C., Pettigrew J.D., Wallis G.M., Hasler F., Vollenweider F.X. (2005). Using psilocybin to investigate the relationship between attention, working memory, and the serotonin 1A and 2A receptors. J. Cognit. Neurosci..

[bib12] Cohen S., Janicki-Deverts D. (2012). ‘Who's stressed? Distributions of psychological stress in the United States in probability samples from 1983, 2006, and 2009’. J. Appl. Soc. Psychol..

[bib13] Corbin N., Todd N., Friston K.J., Callaghan M.F. (2018). Accurate modeling of temporal correlations in rapidly sampled fMRI time series. Hum. Brain Mapp..

[bib14] Cowen A., Sauter D., Tracy J.L., Keltner D. (2019). Mapping the passions: toward a high-dimensional taxonomy of emotional experience and expression. Psychol. Sci. Publ. Interest J. Am. Psychol. Soc..

[bib15] Davis M., Whalen P.J. (2001). The amygdala: vigilance and emotion. Mol. Psychiatr..

[bib16] Davis A.K. (2021). Effects of psilocybin-assisted therapy on major depressive disorder: a randomized clinical trial. JAMA Psychiatr..

[bib17] Demetriou L., Kowalczyk O.S., Tyson G., Bello T., Newbould R.D., Wall M.B. (2018). A comprehensive evaluation of increasing temporal resolution with multiband-accelerated protocols and effects on statistical outcome measures in fMRI. Neuroimage.

[bib18] Dunn O.J. (1961). Multiple comparisons among means. J. Am. Stat. Assoc..

[bib19] Fisher P.M. (2009). Medial prefrontal cortex 5-HT2A Density is correlated with amygdala reactivity, response habituation, and functional coupling. Cereb. Cortex N. Y. NY.

[bib20] Fisher P.M. (2011). Medial prefrontal cortex serotonin 1A and 2A receptor binding interacts to predict threat-related amygdala reactivity. Biol. Mood Anxiety Disord..

[bib21] Fisher P.M., Haahr M.E., Jensen C.G., Frokjaer V.G., Siebner H.R., Knudsen G.M. (2015). Fluctuations in [11C]SB207145 PET binding associated with change in threat-related amygdala reactivity in humans. Neuropsychopharmacol. Off. Publ. Am. Coll. Neuropsychopharmacol..

[bib22] Fisher P.M. (2022). Emotional faces processing in major depressive disorder and prediction of antidepressant treatment response: a NeuroPharm study. J. Psychopharmacol. Oxf. Engl..

[bib23] Fusar-Poli P. (2009). Functional atlas of emotional faces processing: a voxel-based meta-analysis of 105 functional magnetic resonance imaging studies. J. Psychiatry Neurosci. JPN.

[bib24] Garcia-Romeu A., Davis A.K., Erowid F., Erowid E., Griffiths R.R., Johnson M.W. (2019). Cessation and reduction in alcohol consumption and misuse after psychedelic use. J. Psychopharmacol. Oxf. Engl..

[bib25] Griffiths R.R., Richards W.A., McCann U., Jesse R. (2006). Psilocybin can occasion mystical-type experiences having substantial and sustained personal meaning and spiritual significance. Psychopharmacology (Berl.).

[bib26] Griffiths R.R. (2016). Psilocybin produces substantial and sustained decreases in depression and anxiety in patients with life-threatening cancer: a randomized double-blind trial. J. Psychopharmacol. Oxf. Engl..

[bib27] Grob C.S. (2011). Pilot study of psilocybin treatment for anxiety in patients with advanced-stage cancer. Arch. Gen. Psychiatr..

[bib28] Hariri A.R., Bookheimer S.Y., Mazziotta J.C. (2000). Modulating emotional responses: effects of a neocortical network on the limbic system. Neuroreport.

[bib29] Haxby J.V., Hoffman E.A., Gobbini M.I. (2002). Human neural systems for face recognition and social communication. Biol. Psychiatr..

[bib30] Healy C.J. (2021). The acute effects of classic psychedelics on memory in humans. Psychopharmacology (Berl.).

[bib31] Hornboll B. (2013). Acute serotonin 2A receptor blocking alters the processing of fearful faces in the orbitofrontal cortex and amygdala. J. Psychopharmacol. Oxf. Engl..

[bib32] Janak P.H., Tye K.M. (2015). From circuits to behaviour in the amygdala. Nature.

[bib33] Johnson M.W., Hendricks P.S., Barrett F.S., Griffiths R.R. (2019). Classic psychedelics: an integrative review of epidemiology, therapeutics, mystical experience, and brain network function. Pharmacol. Ther..

[bib34] Kometer M., Schmidt A., Bachmann R., Studerus E., Seifritz E., Vollenweider F.X. (2012). Psilocybin biases facial recognition, goal-directed behavior, and mood state toward positive relative to negative emotions through different serotonergic subreceptors. Biol. Psychiatr..

[bib35] Kraehenmann R. (2015). Psilocybin-induced decrease in amygdala reactivity correlates with enhanced positive mood in healthy volunteers. Biol. Psychiatr..

[bib36] LeDoux J.E. (2000). Emotion circuits in the brain. Annu. Rev. Neurosci..

[bib37] Madsen M.K., Knudsen G.M. (2021). Plasma psilocin critically determines behavioral and neurobiological effects of psilocybin. Neuropsychopharmacol. Off. Publ. Am. Coll. Neuropsychopharmacol..

[bib38] Madsen M.K. (2019). Psychedelic effects of psilocybin correlate with serotonin 2A receptor occupancy and plasma psilocin levels. Neuropsychopharmacol. Off. Publ. Am. Coll. Neuropsychopharmacol..

[bib39] Madsen M.K. (2021). Psilocybin-induced changes in brain network integrity and segregation correlate with plasma psilocin level and psychedelic experience. Eur. Neuropsychopharmacol. J. Eur. Coll. Neuropsychopharmacol..

[bib40] McCulloch D.E.-W. (2023). Navigating the chaos of psychedelic neuroimaging: a multi-metric evaluation of acute psilocybin effects on brain entropy. medRxiv.

[bib41] McNabb C.B., Lindner M., Shen S., Burgess L.G., Murayama K., Johnstone T. (2020). Inter-slice leakage and intra-slice aliasing in simultaneous multi-slice echo-planar images. Brain Struct. Funct..

[bib42] Moreno F.A., Wiegand C.B., Taitano E.K., Delgado P.L. (2006). Safety, tolerability, and efficacy of psilocybin in 9 patients with obsessive-compulsive disorder. Dis. Nerv. Syst..

[bib43] Mueller F. (2017). Acute effects of LSD on amygdala activity during processing of fearful stimuli in healthy subjects. Transl. Psychiatry.

[bib44] Muthukumaraswamy S.D., Forsyth A., Lumley T. (2021). Blinding and expectancy confounds in psychedelic randomized controlled trials. Expet Rev. Clin. Pharmacol..

[bib45] Nichols D.E. (2016). Psychedelics. Pharmacol. Rev..

[bib46] Passie T., Seifert J., Schneider U., Emrich H.M. (2002). The pharmacology of psilocybin. Addict. Biol..

[bib47] Plichta M.M. (2012). Test-retest reliability of evoked BOLD signals from a cognitive-emotive fMRI test battery. Neuroimage.

[bib48] Pokorny T., Preller K.H., Kometer M., Dziobek I., Vollenweider F.X. (2017). Effect of psilocybin on empathy and moral decision-making. Int. J. Neuropsychopharmacol..

[bib49] Raines T.C., Reynolds C.R., Kamphaus R.W. (2018). Contemporary Intellectual Assessment: Theories, Tests, and Issues.

[bib50] Rhodes R.A. (2007). Human 5-HT transporter availability predicts amygdala reactivity in vivo. J. Neurosci. Off. J. Soc. Neurosci..

[bib51] Robinson O.J. (2013). The role of serotonin in the neurocircuitry of negative affective bias: serotonergic modulation of the dorsal medial prefrontal-amygdala “aversive amplification” circuit. Neuroimage.

[bib52] L. Roseman, D. J. Nutt, and R. L. Carhart-Harris, ‘Quality of acute psychedelic experience predicts therapeutic efficacy of psilocybin for treatment-resistant depression’, Front. Pharmacol.. . 10.3389/fphar.2017.00974 PMC5776504 29387009

[bib53] Roseman L., Demetriou L., Wall M.B., Nutt D.J., Carhart-Harris R.L. (2018). Increased amygdala responses to emotional faces after psilocybin for treatment-resistant depression. Neuropharmacology.

[bib54] Roseman L., Haijen E., Idialu-Ikato K., Kaelen M., Watts R., Carhart-Harris R. (2019). Emotional breakthrough and psychedelics: validation of the emotional breakthrough inventory. J. Psychopharmacol. Oxf. Engl..

[bib55] Ross S. (2016). Rapid and sustained symptom reduction following psilocybin treatment for anxiety and depression in patients with life-threatening cancer: a randomized controlled trial. J. Psychopharmacol. Oxf. Engl..

[bib56] Said C.P., Haxby J.V., Todorov A. (2011). Brain systems for assessing the affective value of faces. Philos. Trans. R. Soc. Lond. B Biol. Sci..

[bib57] Schmidt A., Kometer M., Bachmann R., Seifritz E., Vollenweider F. (2013). The NMDA antagonist ketamine and the 5-HT agonist psilocybin produce dissociable effects on structural encoding of emotional face expressions. Psychopharmacology (Berl.).

[bib58] Sheehan D.V. (1998). The Mini-International Neuropsychiatric Interview (M.I.N.I.): the development and validation of a structured diagnostic psychiatric interview for DSM-IV and ICD-10. J. Clin. Psychiatry.

[bib59] Stenbæk D.S. (2021). Brain serotonin 2A receptor binding predicts subjective temporal and mystical effects of psilocybin in healthy humans. J. Psychopharmacol. Oxf. Engl..

[bib60] Studerus E., Gamma A., Vollenweider F.X. (2010). Psychometric evaluation of the altered states of consciousness rating scale (OAV). PLoS One.

[bib61] Studerus E., Kometer M., Hasler F., Vollenweider F.X. (2011). Acute, subacute and long-term subjective effects of psilocybin in healthy humans: a pooled analysis of experimental studies. J. Psychopharmacol. Oxf. Engl..

[bib62] Todd N., Moeller S., Auerbach E.J., Yacoub E., Flandin G., Weiskopf N. (2016). Evaluation of 2D multiband EPI imaging for high-resolution, whole-brain, task-based fMRI studies at 3T: sensitivity and slice leakage artifacts. Neuroimage.

[bib63] Vollenweider F.X., Preller K.H. (2020). Psychedelic drugs: neurobiology and potential for treatment of psychiatric disorders. Nat. Rev. Neurosci..

[bib64] Vollenweider F.X., Vollenweider-Scherpenhuyzen M.F.I., Bäbler A., Vogel H., Hell D. (1998). Psilocybin induces schizophrenia-like psychosis in humans via a serotonin-2 agonist action. Neuroreport.

[bib65] Watts R., Day C., Krzanowski J., Nutt D., Carhart-Harris R. (2017). ‘Patients’ accounts of increased “connectedness” and “acceptance” after psilocybin for treatment-resistant depression’. J. Humanist. Psychol..

