# Supplementary material for: Amygdala response to emotional faces following acute administration of psilocybin in healthy individuals
Source: Neurosci Appl. 2023 Dec 30;3:103934. doi: 10.1016/j.nsa.2023.103934 (PMC12244094; doi:10.1016/j.nsa.2023.103934)
Supplement: Multimedia component 1 [file mmc1.docx]

**SUPPLEMENTARY TEXT**

Amygdala response to emotional faces following acute administration of psilocybin in healthy individuals

Sophia Armand^1,2^ Kristian Larsen^1,5^ Martin K. Madsen^1^ Brice Ozenne^1,3^ Katrin H. Preller^4^ Gitte M. Knudsen^1,5^ Dea S. Stenbæk*^1,2^ & Patrick M. Fisher*^1,6^

*Shared senior author

^1^Neurobiology Research Unit and NeuroPharm, Copenhagen University Hospital Rigshospitalet, Denmark

^2^Department of Psychology, University of Copenhagen, Denmark

^3^Department of Public Health, Section of Biostatistics, University of Copenhagen, Denmark

^4^Department of Psychiatry, Psychotherapy and Psychosomatics, University of Zurich, Switzerland

^5^Department of Clinical Medicine, University of Copenhagen, Denmark

^6^Department of Drug Design and Pharmacology, University of Copenhagen, Denmark

**Corresponding author:**

Patrick MacDonald Fisher, Ph.D.

Neurobiology Research Unit, Rigshospitalet,

6-8 Inge Lehmanns Vej, Building 8057, DK-2100 Copenhagen O

Ph: +45 35 45 67 14, mail: patrick@nru.dk, fax: +45 35 45 67 13

Exclusion criteria

The exclusion criteria were: 1) past or current primary psychiatric disease (DSM axis 1 or WHO ICD-10 diagnostic classifications) or in first-degree relatives, 2) past or current neurological disease or significant somatic disease, 3) past or current substance or drug abuse, 4) non-fluent Danish language skills, 5) vision or hearing impairment, 6) past or current learning disability, 7) intake of drugs suspected to influence test results, 8) allergy to test drugs, 9) intake of QT-prolonging medication or electrocardiogram (ECG) results indicative of heart disease, 10) blood donation less than three months before project participation, 11) bodyweight lower than 50 kg, 12) low plasma ferritin levels (< 12 μg/L), 13) pregnancy or breastfeeding, 14) MRI contraindications, 15) significant exposure to radiation within the past year.

Pre-processing of fMRI data

Functional images were pre-processed and analysed in SPM12. Single-subject functional volumes were corrected for slice-timing (MRI_1_ data only), unwarped and realigned to a subject-specific mean functional image. Functional images were smoothed using a 4 mm FWHM Gaussian filter. Functional images were kept in subject space for analysis due to our focus on regional effects and we therefore sought to limit image modulation (e.g., interpolation). The Automatic Anatomical Label (AAL) atlas was used to define brain regions.^53^ The AAL atlas was warped into subject-space by co-registering the high-resolution T1-weighted structural image with the functional images. We then normalised the T1-weighted image into Montreal Neurological Institute (MNI) standard space and applied the inverse warping map to the AAL atlas in MNI space (using nearest neighbour interpolation to maintain region labels) to generate an AAL atlas in subject space. The subject-space AAL atlas was resliced to match the voxel dimensions of the functional images. Only the 90 cortical/subcortical regions were included in analyses. We used the artefact detection tool (ART; https://www.nitrc.org/projects/artifact_detect/) to identify individual functional volumes with excess motion (>2mm) and signal variability (>4 SD).

**Supplementary Figure Lengend**

**Supplementary Figure 1.** Illustration of the emotional faces paradigm during fMRI. Participants are instructed to match the target stimuli (in the top) to one of the two potential matching stimuli on the bottom left and right sides as fast and accurately as possible using a hand controller pressing the left or right key. The entire paradigm consists of four blocks of emotional faces (i.e., fearful faces, angry faces, surprised faces and neutral faces presented in random order across four versions) interleaved by five control blocks consisting of geometric shapes. Within each block, six face trios were displayed for four seconds, each interleaved by a fixation cross displayed with variable time intervals (i.e., two, four or six seconds) to minimise expectancy effects and habituation while maximising amygdala response throughout the paradigm. For shapes, a fixation cross was displayed with a fixed time interval of two seconds, interleaving each of the six shape trios. The paradigm takes a total of 6.5 minutes to complete. B. In this study, we only attended to negative facial expressions (i.e. anger and fear) and neutral faces, as well as geometric shapes, which were used to contrast each facial expression.
